# The impact of a 1 mm interimplant distance on the interproximal crestal bone height: a case report with a 10-year follow-up and literature review

**DOI:** 10.1186/s40729-025-00589-8

**Published:** 2025-02-01

**Authors:** David Morales Schwarz, Serge Szmukler-Moncler, Hilde Morales Melendez, Florian Beuer

**Affiliations:** 1 Clinica Dental M&M, Valladolid, Spain; 2 Department of Prosthodontics, Charité University of Medicine, Berlin, Charité Center 03, Assmannshauser Str. 4-6, 14197, Berlin, Germany; 3 Berlin Implantology Research Group, Eichhornstrasse 02, 10785 Berlin, Germany

**Keywords:** Review, Case report, Interimplant distance, Crestal bone loss, Papilla, Conical connection, Platform switching

## Abstract

**Background:**

Between adjacent dental implants, an interimplant distance (IID) of at least 3 mm has been recommended to avoid resorbing the interproximal crestal bone. The effect of a 2 mm IID on crestal bone loss has been investigated but the literature is scarce when it comes to an IID of 1 mm. There is a need to document such clinical situations when they occur and elucidate if such a narrow IID is deleterious or not to the interproximal crest. The present case deals with an IID of 1 mm in the premolar area where, for the first time, the fate of an interimplant crest is reported after a 10-year follow-up.

**Case presentation:**

A 57-year-old patient attended with 2 hopeless maxillary premolars. The mesio-distal space available for implant rehabilitation was too narrow to receive standard diameter implants and keep an inter-implant distance (IID) of 3 mm as recommended by accepted guidelines. A protocol of immediate implant placement and provisionalization involving 2 implants of Ø 3.5 mm was implemented; placement in the extraction sockets resulted in an IID of 1 mm. After 3 months of healing the final prosthesis was delivered; the patient has been followed for 10 years now. Surprisingly, the findings showed that the interimplant crest was maintained 1.40 mm coronal to the shoulder of the neck of the implants. Bone completely filled the space between the prosthetic concave abutments and the interproximal papilla was closing the embrasure. The literature reports only 2 experimental studies involving a 1 mm IID; both showed that this did not lead to the resorption of the interproximal bone.

**Conclusions:**

Unexpectedly, the present case with an IID of 1 mm did not lead to the resorption of the interproximal bone after 10 years. It is speculated that the reason for that is due to the implants displaying an internal conical connection, the platform-switching feature, concave abutments and subcrestal placement. The fate of the interproximal crest of implants placed with an IID of 1 mm lacks scientific evidence. More studies are warranted to elucidate this question in order to propose the best implant treatment in cases displaying a limited mesio-distal space.

## Background

Implant therapy is presently a routine and well-established treatment modality for the rehabilitation of missing teeth. A key measure of long-term clinical success is the maintenance of bone at the shoulder of the implant [1, 2].

3D implant placement in the oro-facial [3], mesio-distal [4] and corono-apical directions [5] have been identified as critical parameters regulating the peri-implant crestal bone loss. More specifically, in the mesio-distal direction, recommendations currently exist on a minimum distance of 1.5 mm between an implant and an adjacent tooth and at least 3 mm between 2 adjacent implants [4, 6–8].

Two decades ago, the issue of keeping at least 3 mm apart between 2 adjacent implants have been raised by Tarnow et al. [6, 7] after observation of a loss of the interimplant bone crest when this distance was not respected. Many others authors corroborated the correlation between IID inferior to 3 mm and bone loss [9–17]. This observation was done with implants having an external hex connection and a matching between the implant collar and the abutment. In the meantime, the literature investigated the merit of this recommendation for implants displaying the platform-switching feature and an internal conical connection. Several animal [18–25] and clinical [26, 27] studies took it at task and compared the bone response to an interimplant distance (IID) of 2 mm vs. the recommended 3 mm. Rarely however, the IID was less than 2 mm [16, 18, 28].

Noteworthy, the issue of losing crestal bone at the immediate vicinity of the implant-abutment junction (IAJ) described by Tarnow et al. [6, 7] was related to implants involving a gap at the IAJ placed at the level of the crest. The latter harbors bacteria that are leading to a localized chronic inflammation [29, 30] and to the subsequent bone loss that has been described by Tarnow et al. [6, 7]. More recent terminology calls bone level (BL) implants those implants with the IAJ situated at the vicinity of the crest and tissue level (TL) implants those implants with the IAJ located at the gingival level ([31, 32]. Hermann et al. [29] investigated the bone resorption that is occurring around BL and TL implants in a non-loaded model in the mandible of the dog; they found that crestal bone loss was significantly less for the TL design compared to the BL one; in addition, this difference was noticeable yet after 6 months of implantation, even before prosthetic loading. A similar result was identified on sites having undergone vertical bone augmentation; after a mean loading time of 33 months; the bone loss measured at the TL implants was inferior to the one of the BL group [31]. Moreover, in a 9-year follow-up study on Swedish patients, Derks et al. [33] found that the prevalence of peri-implantitis was lower for TL implants when compared to BL ones; the odd ratio was 3.55–5.56 for the BL implants. These findings might suggest that TL implants that are lacking a gap might be less concerned by the biologic issue raised by Tarnow et al. [6, 7].

The aim of the present paper is to present a case of BL implants with a 1 mm IID in the premolar area of the maxilla that has been followed for 10 years, while reviewing the scientific literature of BL implants exposed to such a narrow IID. This IID is much less than the recommended IID and accordingly, the interimplant crest was expected to resorb in line with the recommended guidelines [4, 6–8]. Yet, follow-up of this patient showed that the interimplant crest was maintained up to the 10-year recall. The case is discussed as to understand what could be the reasons the interimplant crest was maintained over time and why the foreseen bone loss between the 2 implants separated by 1 mm only [6, 7] didn’t occur.

To review the existing scientific and clinical literature on this topic, articles published until July 2024 in the MEDLINE- PubMed database were searched. For the sake of the broadest quest, the free text term (“interimplant distance”) was searched in the database. The initial electronic result identified 161 references; however, many references were unrelated to the topic, they were dealing with prosthetic biomechanical issues and the precision of intra-oral scanning. Therefore, the search was further refined to “interimplant distance” AND “bone”. The term “bone” is more inclusive than ‘bone loss’ or ‘bone resorption’; the reason was to avoid missing any reference using another locution for bone loss. Ninety-one references were identified; retrieval of the abstracts and their evaluation led to select 37 full-text articles.

**Fig. 1:**
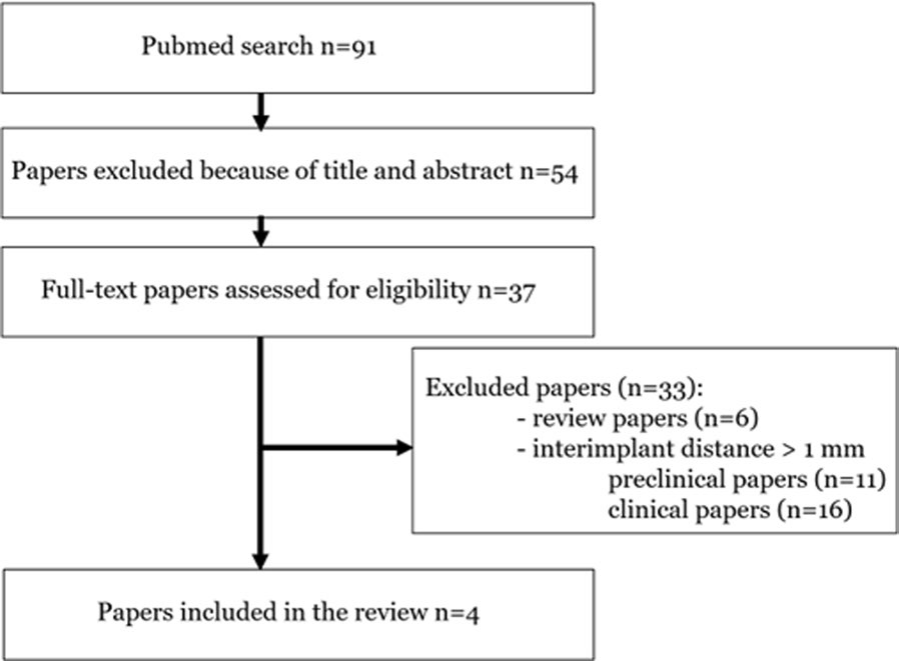
Chartflow of the literature search

The papers were distributed into 15 preclinical studies, 16 clinical references and 6 reviews of the literature. Most of the studies were comparing the standard recommended IID of 3 mm to an IID of 2 mm. No clinical study or report focused on the 1 mm IID; a single paper dealt with an IID inferior to 2 mm [16], however, detailed information on this variable were not provided. Finally, only 4 experimental papers were identified for inclusion in the review [18, 21, 22, 28]. Three of them shared the same protocol and animals [18, 21, 22]; each one investigated a distinct bone variable of the same in vivo study. The search process is shown on Fig. 1; Table 1 lists the included references and the characteristics of the studies.

**Table 1:**
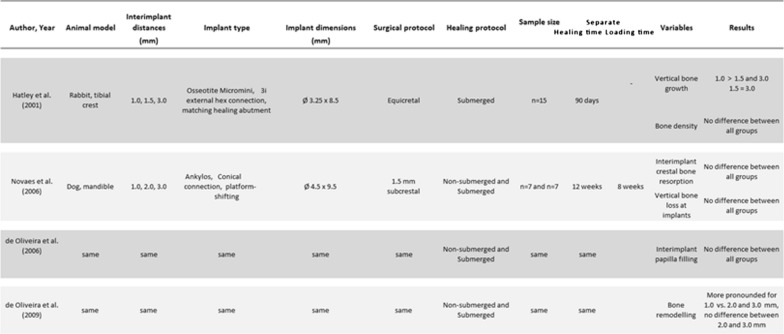
Characteristics of the 4 published papers found in the search

In reality, only 2 animal studies have been performed; the second preclinical experiment had its results published in 3 different papers, each one focusing on a distinct bone variable

Thus, only 2 animal studies have focused on the 1 mm IID topic and compared this narrow IID to larger ones. One was performed in the tibial crest of the rabbit [28]; the study compared the bone response to implants displaying an IID of 1 mm, 1.5 mm and 3.0 mm after a submerged healing of 90 days. The implants of this study presented an external hex connection and matching cover-screws [28]. The other was caried out in the dog mandible and was mimicking the reality of functional loading [18, 21, 22]; the distance between the implants were 1 mm, 2 mm and 3 mm. The implants had a conical internal connection and the platform switching feature; they were left to heal for 12 weeks and were further loaded for 8 weeks [18, 21, 22]. Surprisingly, both studies reported better bone response features for the 1 mm IID when compared to larger ones.

Table 1 details the investigated variables of the studies and the corresponding results. The rabbit study [28] analyzed the bone density between the implants and the related vertical bone growth. Bone density showed no difference between the various IIDs. Vertical bone growth was superior for the 1 mm IID compared to the 2 and 3 mm IIDs; however, no difference was noted for this variable when the 2 mm IID was compared to the 3 mm IID that is recommended by the guidelines. The loaded experiment in the dog mandible compared, between the 3 groups, the interimplant crestal bone resorption [18], the vertical bone loss at the implants [18], the papilla filling of the interimplant embrasure [21] and the bone remodelling between the neighbouring implants [22]. Only the bone remodeling activity was different between the 3 groups of IID; the 1 mm was superior to the 2 other larger IIDs while no difference were found between the 2 mm and 3 mm IIDs. All other variables were identical, especially the interimplant crestal bone resorption.

## Case presentation

The report of this case follows the CARE guidelines. The patient was treated according to latest Helsinki declaration and an informed consent was signed.

### Patient

**Fig. 2:**
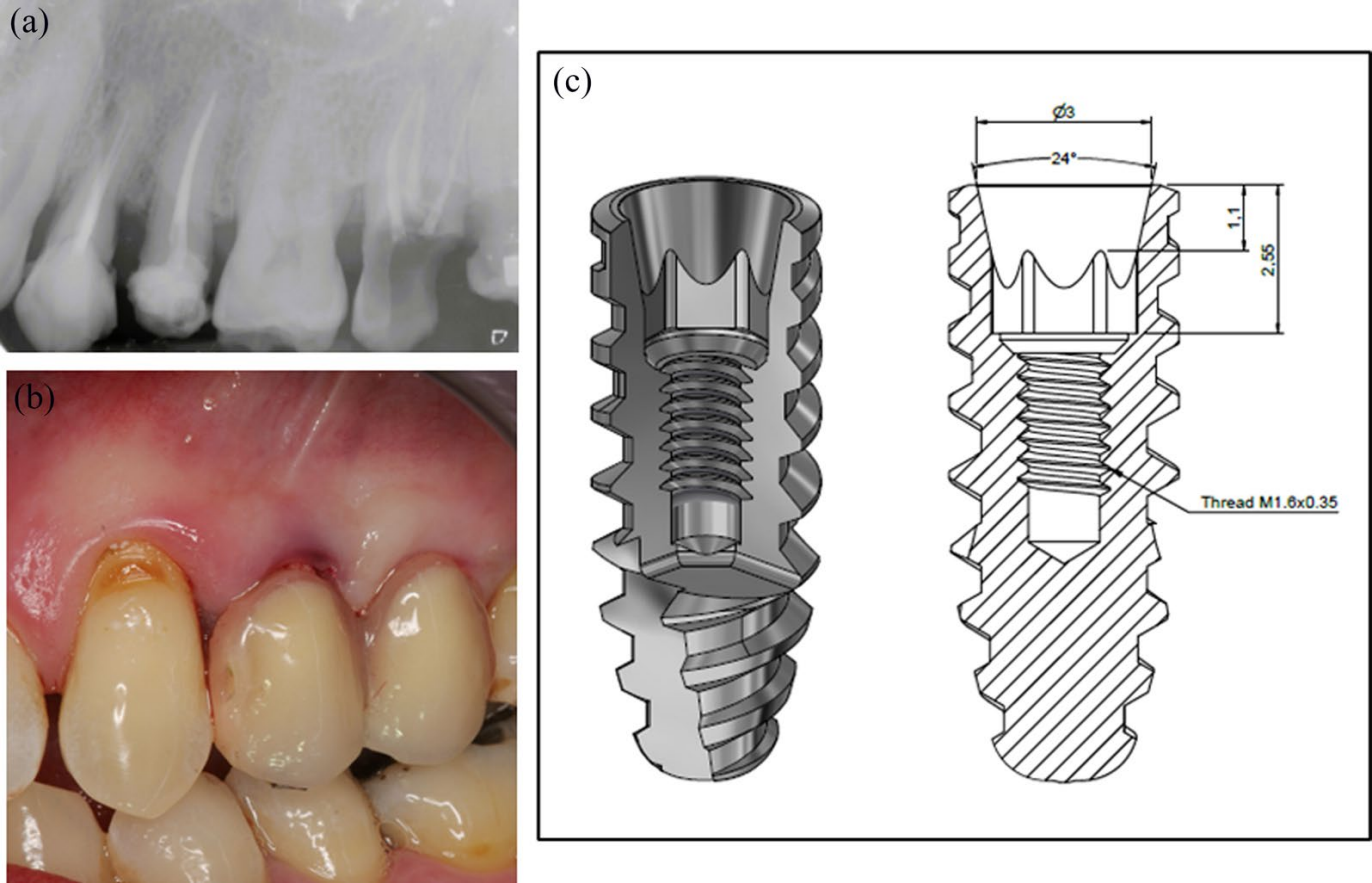
Preoperative and postoperative situation. **a** Periapical taken when the patient attended the anamnesis. Note the black apical image at the root of tooth #24 and the fractured crown of tooth #25**. b** Characteristics of the implant used to treat this patient. Note the cylindroconical shape, the 24-degree internal conical connection with its hexagonal index and the in-built 0.25 mm platform-shifting. **c** Vestibular view of the treated sites upon delivery of the temporary prosthesis. The shape of the crowns is esthetical despite the narrow interimplant distance

A 57-year-old female patient attended with a chief complain of pain related to tooth #24 and fracture of the crown of the second left premolar. Clinical observation showed pain upon palpation of tooth#24 and partial loss of the crown of tooth #25. Panoramic and periapical radiologic examination revealed the presence of an endodontic treatment on both teeth, an apical lesion on tooth # 24 and a root fracture of tooth #25 (Fig. 2a).

Both teeth were considered hopeless and required extraction; the patient requested an immediate esthetic solution. Implant therapy was considered and a protocol of tooth extraction, immediate implant placement and immediate provisionalization was contemplated. Soft tissue analysis showed a low smile line and a thick gingival biotype.

Patient was smoking more than 10 cigarettes a day, general health was noncontributory but dental hygiene was deficient. Advanced decay was observed on several endodontically treated teeth; the left canine showed signs of abrasion at the dental neck and a consequent recession of the marginal gingiva.

The available mesio-distal space following extraction of the teeth was 12 mm. Application of the current rules of 3D implant positioning in the horizontal axis [4, 6–8] would require the placement of 2 standard adjacent Ø 3.75 mm implants, keeping an interimplant distance of at least 3 mm and maintaining each implant at least 1.5 mm apart from the adjacent teeth, i.e. a total of at least 13.5 mm. Implementing the root submergence technique described by Salama et al. [34] or placing a single standard implant with a crown in extension [35, 36] was considered in order to avoid the 2 adjacent implants configuration that is supposed, according to the literature, to lead to the loss of the inter-implant crestal bone [4, 6–8] and jeopardize the interproximal papilla [37]. However, these solutions were discarded, first because the additional forces and moments, that would be exerted by the immediate provisionalization on the single implant placed in a post-extraction socket in the posterior area, might jeopardize its osseointegration; second, because of the long-term biomechanical concern of such a prosthetic protocol in the posterior area [35, 38]. Therefore, placement of 2 adjacent narrow Ø 3.50 mm implants made of titanium grade 23, the strongest available biocompatible titanium grade, was decided. Keeping the minimal 1.5 mm distance between the implants and the adjacent healthy teeth was prioritized.

### Treatment of the case

A preoperative impression was taken in order to prepare the shell of the temporary prosthesis; subsequently, atraumatic extraction of the 2 premolars was performed. Two cylindroconical implants of Ø 3.50 × 13 mm (Fig. 2b) were placed in an approximately 1.5 mm infra-osseous position with regard to the vestibular table with an insertion torque superior to 35 Ncm. Insertion of the 2 implants in the post-extraction sockets with sufficient primary stability to withstand an immediate provisionalization protocol resulted in a 1 mm IID measured between the implant collars; this is much less than the recommended 3 mm IID [4, 6–8, 37].

The placed implants (Top DM, Bioner, San Juan Desvest, SP) are made of titanium grade 23 with an inbuilt 0.25 mm platform-shifting feature and a hexagonally indexed 24° internal conical connection (Fig. 2b). The surface presents a regular pattern of macro- and micropores that has been obtained by etching only (BioEtch^®^) without sandblasting [39] up to the top and bevel of the collar. The remaining room in the post-extraction sockets, between the walls and the implants, was filled with a PRP platelet rich fibrin clot without any bone substitute. Subsequently, 2 multi-unit abutments (Micromini Ø 3.50 mm, Bioner, San Juan Desvest, SP) with a concave gingival profile of 0.3 mm were affixed to the internal conical connection of the implants; the gingival height of the abutments of the first and second premolar were 1.5 and 2.5 mm, respectively. After suturing the sockets, provisional copings made of titanium were placed on top of the Micromini abutments. During surgery, a 2-unit acrylic temporary prosthesis was prepared by the laboratory based on the preoperative impression; it was relined and trimmed. The screw-retained provisional prosthesis was fastened with a 20Ncm torque (Fig. 2c); adjustments were made to bring the crowns out of occlusion.

Finally, the patient left the office with a provisional implant-supported prosthesis following an immediate implant placement and provisionalization protocol.

### Follow-up of the case

**Fig. 3:**
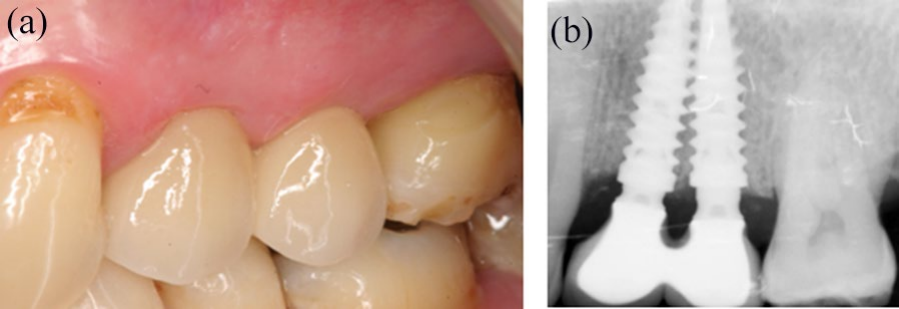
Delivery of the final prosthesis after 3 months of provisionalization. **a** Vestibular view of the prosthesis and the marginal gingiva. The interproximal papilla is closing the embrasure of the ceramo-metallic crowns. **b** Periapical radiography control after placing the final prosthesis. Note the platform-shifting, the concave prosthetic abutments and the interimplant bone that still lacks maturation

**Fig. 4:**
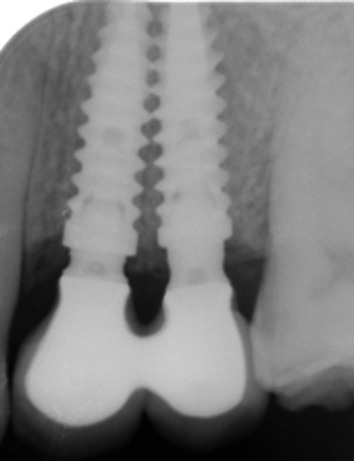
Periapical examination at the 1-year control. Note the densification of the interimplant bone

**Fig. 5:**
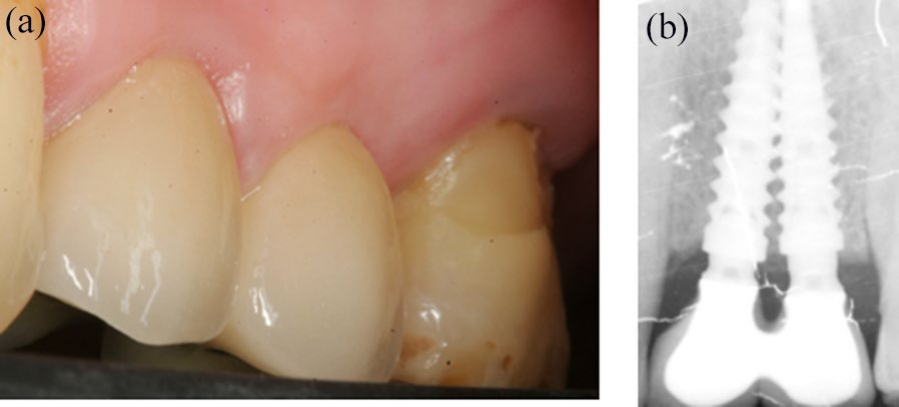
Three-year control. **a** Vestibular view of the implant-supported rehabilitation. The gingiva looks healthy and the interimplant papilla has grown coronally compared to the previous 3-month control. **b** Periapical radiography. The interproximal bone is stable

After 3 months of healing, implant stability was checked clinically and radiographically. A splinted screw-retained 2-unit prosthesis was chosen for definitive rehabilitation because of its superior prognosis compared to 2 non-splinted crowns [40, 41]. Figures 3a and b are showing the final implant-supported porcelain fused to metal fixed dental prosthesis that was delivered. Clinical observation showed the presence of the interproximal papilla; the papillae with the adjacent teeth were partially filling the embrasures (Fig. 3a). The periapical radiography revealed the presence of immature bone in a slightly coronal position to the adjacent implant necks (Fig. 3b). At the 1-year radiographic control, the interimplant crestal bone was denser than at 3 months, the coronal limit was well above the implant-abutment connection; it was completely filling the enlarged space between the concave prosthetic abutments (Fig. 4). At 3 years, the interproximal papilla was present and completely filling the embrasure of the adjacent implant-supported crowns (Fig. 5a); the interimplant bone crest was stable compared to the 1-year control (Fig. 5b).

**Fig. 6:**
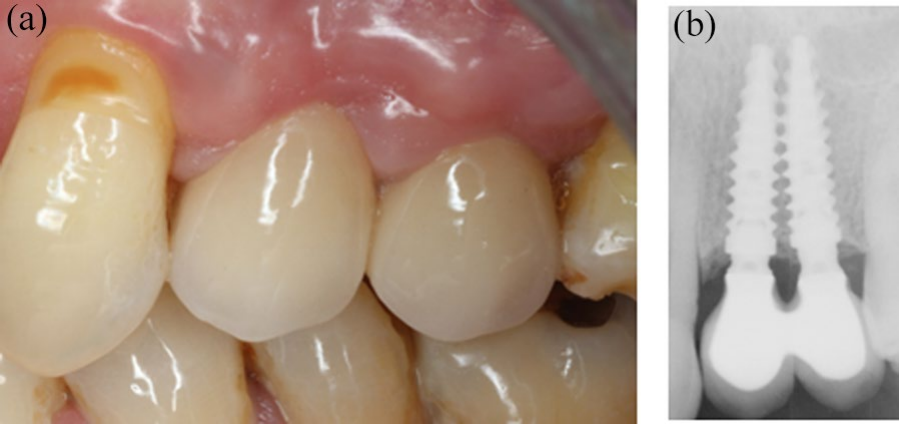
Nine-year control. **a** Vestibular view of the implant-supported rehabilitation. Note the recession observed at the interimplant papilla and the proximal ones. **b** Periapical radiography. The interproximal bone is still covering the implant-abutment connection but seems to have lost some coronal height

During several years the patient was lost to control but she could be reached for the 9-year recall. By that time, the interproximal papilla suffered a noticeable recession but still the interimplant embrasure remained completely filled; this was not the case for the mesial and distal implant-teeth embrasures (Fig. 6a). The interimplant bone was still present but a slight apical resorption seemed to have happened compared to the 3-year control; nevertheless, bone was still present well above the implant-abutment connection of both implants and covering it (Fig. 6b). Hygienic prophylaxis was performed and the smoker patient’s awareness to hygiene was stimulated.

**Fig. 7:**
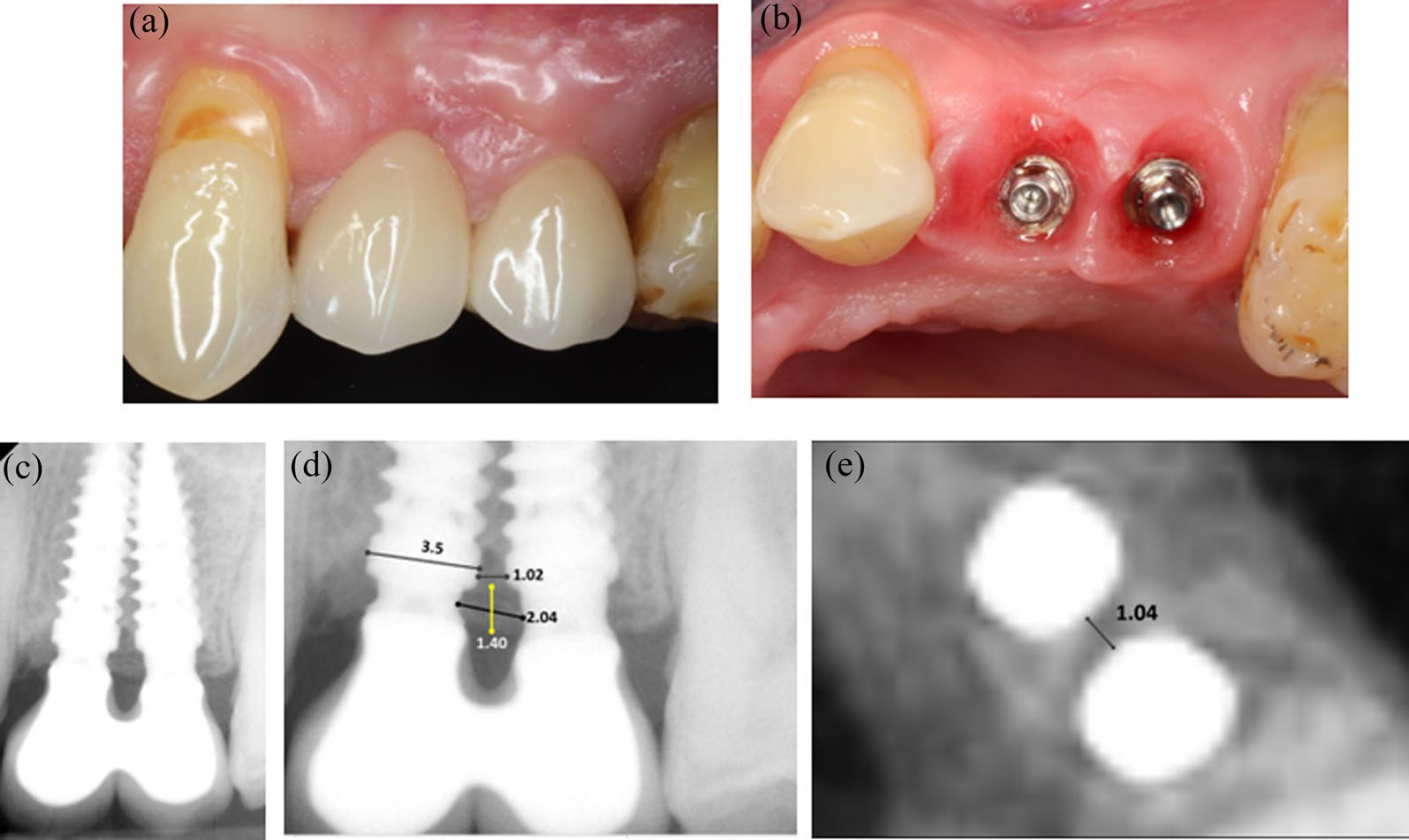
Ten-year control. **a** Vestibular view of the prosthesis relying on 2 close implants with an IID of 1 mm. The interimplant papilla and the proximal ones reached a more coronal position compared to the 9-year recall. **b** Occlusal view of the marginal gingiva after removal of the prosthesis. The soft tissues are looking healthy. **c** Periapical radiography. The interproximal bone is occupying the entire space between the concave abutments; it is more apical compared to the 9-year control. **d** Dimensions at the interimplant area. The implant neck served as internal calibration. Note the distance between the collars of the implants, the overall distance between the concave abutments and the height of the interproximal bone. **e** Measurement of the interimplant distance on the axial axis of the CBCT. The 1.04 mm reading confirms the 1.02 mm IID evaluation on the periapical radiography

One year later, at the 10-year control, the gingiva appeared healthier; all papillae regained height but only the interproximal embrasure was completely filled (Fig. 7a). Table 2 provides the PES/WES evaluation [42] of the implant-supported crowns. The prosthesis was unscrewed to check the peri-implant gingiva; the soft tissues appeared healthy without inflammation (Fig. 7b). On the periapical radiography, the crestal bone levels were well above the implant-abutment connection; the coronal fill of the interimplant space delimited by the concave prosthetic abutments was superior to the previous year (Fig. 7c). Figure 7d shows the various dimensions read on the periapical radiography, the interimplant distance of 1.02 mm, the 2.04 mm room between the concave prosthetic abutments and the 1.40 mm of crestal bone above the implant-abutment connection. At the 10-year control a CBCT examination was also performed before removing the screw-retained prosthesis; the axial section validated the IID measured on the periapical radiography between the collars (Fig. 7e).

**Table 2:**
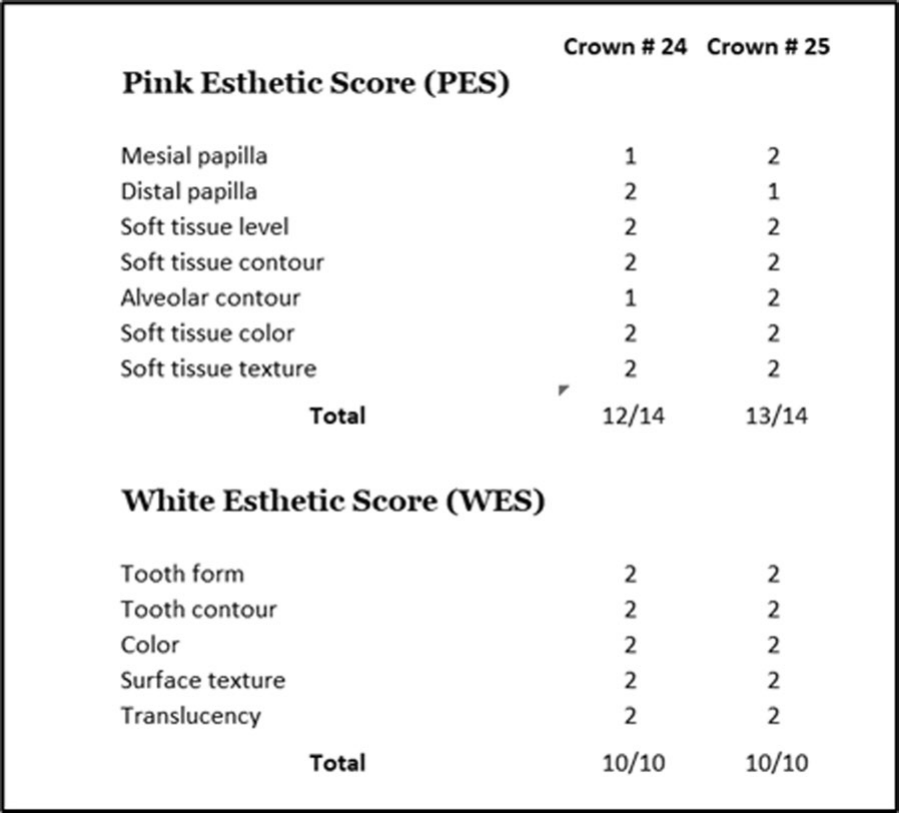
PES and WES scores of the 2 implant-supported crowns at the 10-year recall according to Fürhauser et al. [42]

## Discussion

Respecting the recommended rules of 3D implant placement are considered of paramount importance because they are believed to govern the healthy maintenance of the peri-implant hard and soft tissues over time [4, 6–8, 37]; they are supposed to condition the long-term success of implant therapy [4, 37].

The mesio-distal guidelines have been established 2 decades ago by Tarnow et al. [6, 7] for bone level implants when the standard implant-abutment connection was the external hex without the platform-shifting feature and they are still advocated [4, 37]. Several clinical studies confirmed the relationship between bone loss and an IID of less than 3 mm [11–17], however, these BL implants did not incorporate the contemporary implant-abutment connection which is internal with a conical seal and involves platform-shifting by design. Therefore, there might be some room to question the relevance of these recommendations [4, 37] that have been based on previous outdated BL implant designs. The field of implantology has been through several similar evolutions, e.g. when immediate loading protocols have been advocated against the then paradigm in force [43] and became routinely implemented as a first therapeutic choice or when implantation in infected post-extraction sites once avoided started to be implemented [44].

Several animal and clinical studies compared the bone reaction to a 2 mm IID vs. 3 mm; they repeatedly demonstrated that a 2 mm IID was not detrimental to the interimplant crestal bone. The implants that led to this conclusion had all the platform-shifting and internal connection features [18–27, 45–47]. It is speculated that the reason why only few investigations dealt with the 1 mm IID is most probably due to a lack of clinical relevance owing to the prosthetic limitation of handling correctly the soft tissues within such a limited space [4, 37].

Nonetheless and unexpectedly, the only 2 animal experiments that have compared the 1 mm IID to larger ones came both to the similar conclusion that bone response was superior for this narrow distance as detailed in Table 1. The first one [28] involved implants with an external hex and without the platform-switching feature; however, healing was left submerged. The second [18, 20, 21] implemented contemporary BL implants with internal conical connection and platform-switching and a loading period of 8 weeks. Noteworthy, the results of the present case report with an IDD as narrow as 1 mm is in line with these scarce in vivo studies.

Several parameters related to implant design are affecting the marginal bone levels. Among them are the surface topography at the neck level [48, 49], the presence of a platform-shifting feature [50, 51], the characteristics of the implant-abutment connection [52] and the design of the prosthetic abutment [53, 54].

The BL implant system chosen to treat the present case displays a medium roughened macro- and microstructured surface; this regularly textured surface stretches up to the bevel of the collar (Fig. 2b) and induces a micromechanical anchorage [39]. Its platform-shifting is 0.25 mm; the internal conical connection is 24° over a length of 1.1 mm and it is hexagonally indexed over 1.45 mm. The internal conical connection is one of the safest connections in terms of bone preservation according to our current understanding of prevention of marginal bone loss [52]. In addition, the prosthetic abutments are concave with a 0.3 mm mismatch compared to straight ones. This means that the implants combined some optimal characteristics to retain the crestal bone over the implant shoulder.

The presence of crestal bone filling the entire room between the concave prosthetic abutments was surprising; it might suggest that the implant-abutment connection of the implant is stable and inflammation free, or at least sustains a low inflammation intensity that is compatible with local bone maintenance. Shaping 2 esthetically-looking crowns with an adequate embrasure in such a limited space is challenging; it might be that divergence of the implants alleviated the task.

The present report deals with a single case and obviously no conclusion can be drawn from it; however, the case is interesting for several reasons. First, the period of provisionalization in the post-extraction sockets of the premolar area of the maxilla lasted 3 months only instead of 4 [55] or 6 months [56]; this is similar to implants with surfaces that foster implant osseointegration [57]. This implant design and surface was previously reported to be suitable for early loading protocols in healed ridges [39]; however, no information was available on immediate rehabilitation of post-extraction sockets in the maxilla and on the time of provisionalization. Second, it shows that a proper hard and soft tissue maintenance might be reached over the long-term even when the IID is 1 mm. This degree of proximity between the implants reduces the space available for the interproximal papilla to form properly, thereby most probably affecting adversely the aesthetic result. It might be that an appropriate strategy in this narrow space is to place diverging implants instead of parallel ones in order to ease the realization of esthetic crown units in such a limited room. Third, recession of the papillae and partial resorption of the interimplant crest were observed at the 9-year recall due to a lack of proper hygiene; however, it could be reversed one year later when a more dedicated hygiene was maintained by the patient. Similar tissue recovering have been documented when the irritation factors were removed [58–60]. Fourth, 2 narrow implants of Ø 3.50 mm made of Titanium grade 23 were able to sustain 2 premolars under the much more demanding biomechanical environment than the anterior area.

There is a literature gap regarding the fate of the interproximal crest when the IID is 1 mm for contemporary BL implants with conical internal connection and platform-switching; therefore, experimental and clinical studies are warranted. For evident ethic reasons because of the presently accepted guidelines [4, 37], prospective clinical studies implementing the 1 mm IID in a cohort of cases should be undertaken only after publication of a sufficient number of retrospective cases having documented that the foreseen bone loss is not systematically occurring as suggested. Publication of more similar case reports or cohort of cases should be welcomed by the scientific community to strengthen the evidence on one side or on the other. Finally, the issue of smaller IID should also be extended to TL implants and compare their results to BL implants.

## Conclusions

The fate of the interproxinal crestal bone when the IID of BL implants is 1 mm is lacking in the literature and requires documentation. Therefore, the guidelines of keeping at least 3 mm between 2 implants should still apply to nurture the best conditions of keeping the interimplant crestal bone and supporting the interproximal papilla. Surprisingly however, the only 2 papers that focused on this issue have both reported that an IID of 1 mm, inferior to the recommended 3 mm IID, does not necessary lead to a loss of the interimplant crestal bone.

As to the reported case, the authors speculate that maintaining a crest of 1.40 mm coronal to the implant-abutment collar over 10 years was made possible probably because of the initial infra-crestal position of the implant necks, the presence of a platform-switching feature, the roughened surface reaching the top of the collar, the mechanical stability of the internal conical connection under premolar load and the enlarged space provided by the concave prosthetic abutments.

## Data Availability

No datasets were generated or analysed during the current study.
